# Supplementary material for: Enhancement of the Anticancer Ability of Natural Killer Cells through Allogeneic Mitochondrial Transfer
Source: Cancers (Basel). 2023 Jun 17;15(12):3225. doi: 10.3390/cancers15123225 (PMC10296914; doi:10.3390/cancers15123225)
Supplement: Supplementary file 1 [file cancers-15-03225-s001.zip › cancers-2298934-supplementary.pdf]

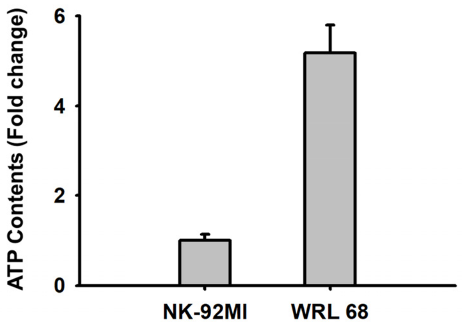

Figure S1. Comparison of the ATP contents of NK-92MI and WRL-68 cells.

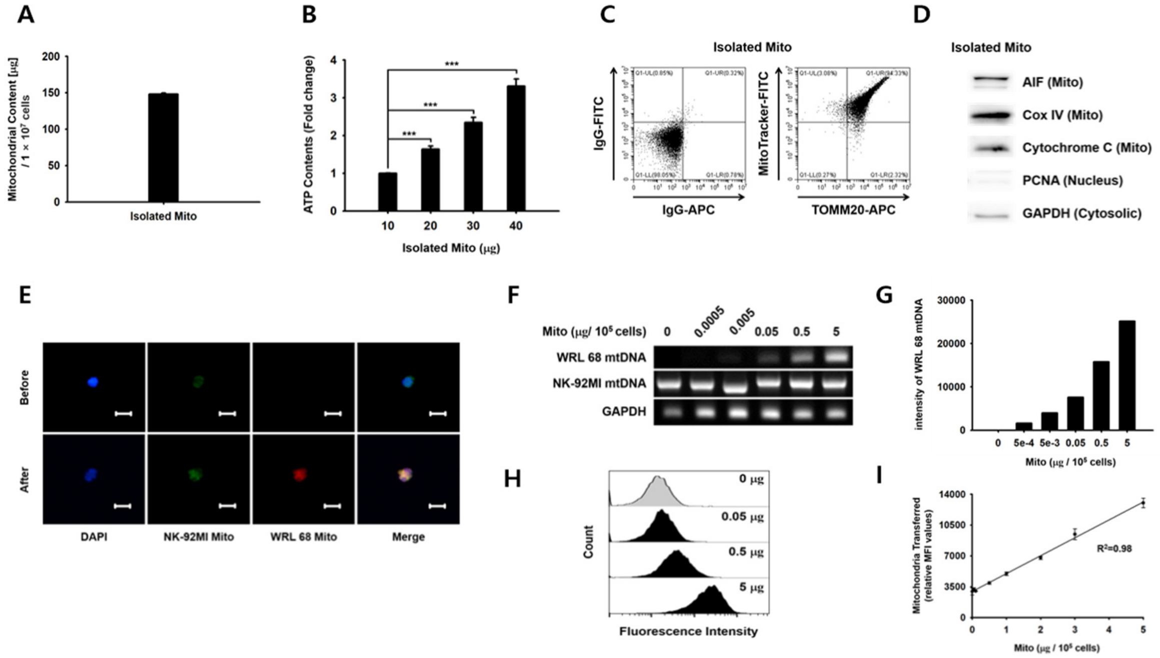

Figure S2. (**A–I**) Characterization of the mitochondria isolated from WRL-68 cells.

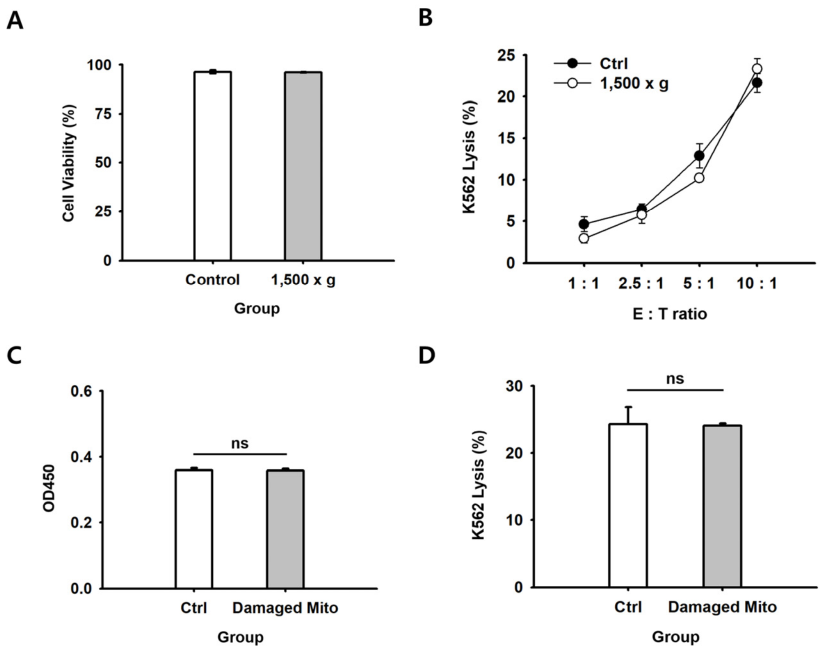

Figure S3. (**A–D**) The enhancement of the anticancer ability of NK-92MI cells is independent of centrifugal stimulation or the presence of damaged mitochondria.

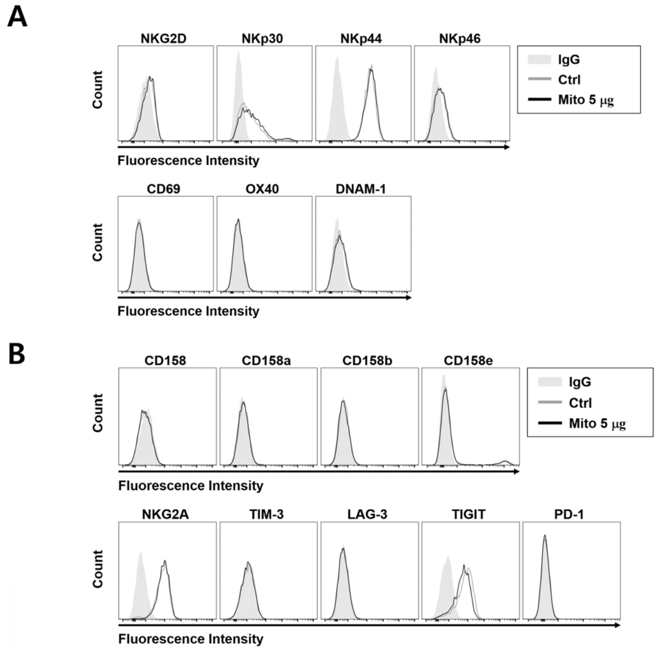

Figure S4. (**A**,**B**) Phenotypic analysis of NK-92MI cells 48 h after mitochondrial transfer.

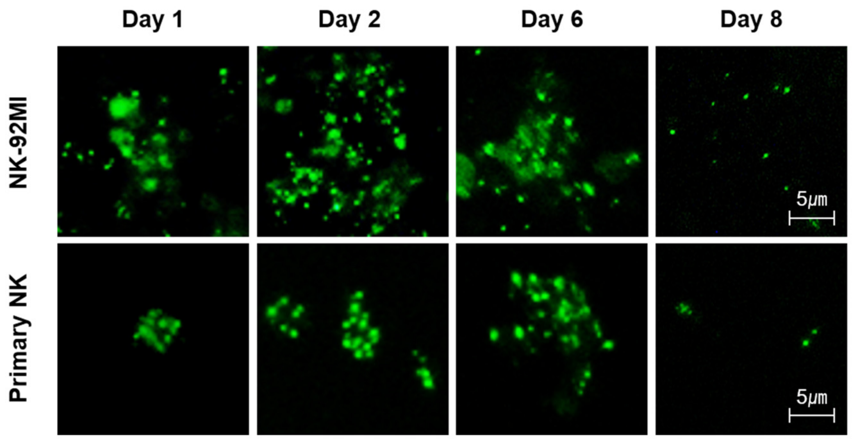

Figure S5. Stability of the transferred mitochondria.

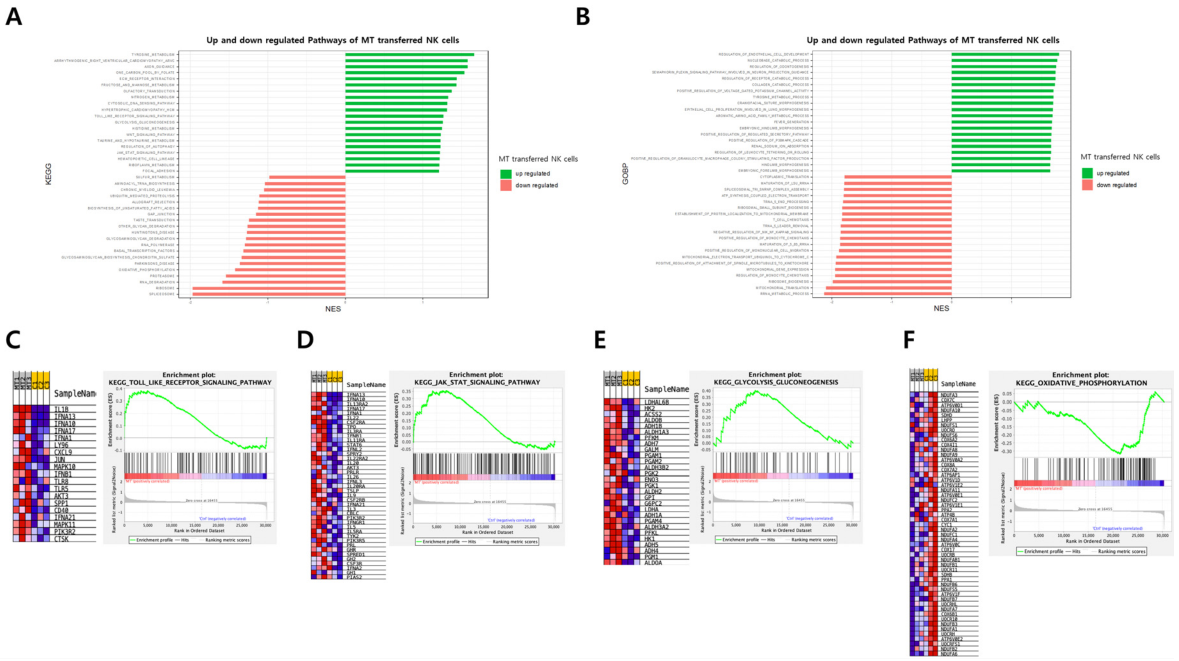

Figure S6. (**A–F**) Changes in gene expression profiling of NK-92MI cells after mitochondrial transfer.
